# Bioengineering hybrid artificial life

**DOI:** 10.3389/fbinf.2025.1676359

**Published:** 2025-12-16

**Authors:** Innocent Sibanda, Geoff Nitschke

**Affiliations:** Department of Computer Science, University of Cape Town, Cape Town, South Africa

**Keywords:** Artificial life, sythetic biology, directed evolution, fitness landscape, evolutionary algorithms

## Abstract

The goal of bioengineering in synthetic biology is to redesign, reprogram, and rewire biological systems for specific applications using standardized parts such as promoters and ribosomes. For example, bioengineered micro-organisms capable of cleaning up environmental pollution or producing antibodies *de novo* to defend against viral pandemics have been predicted. Artificial Life (ALife) facilitates the design and understanding of living systems, not just those found in nature, but *life as it could be*, while synthetic biology provides the means to realize *life as it can be engineered.* Despite significant advances, the synthesis of evolving, adaptable, and bioengineered problem-solving ALife has yet to achieve practical feasibility. This is primarily due to limitations in directed evolution, fitness landscape mapping, and fitness approximation. Thus, currently synthetic (biological) ALife does not continue to evolve and adapt to changing tasks and environments. This is in stark contrast to current digital based ALife that continues to adapt and evolve in simulated environments demonstrating the dictum of *life as it could be*. We posit that if the bioengineering (on-demand design) of problem solving ALife is to ever become a reality then open issues pervading the directed evolution of synthetic ALife must first be addressed. This review examines open challenges in directed evolution, genetic diversity generation, fitness mapping, and fitness estimation, and outlines future directions toward a hybrid synthetic ALife design methodology. This review provides a novel perspective for a singular (hybridized) evolutionary design methodology, combining digital *(in silico)* and synthetic *(in vitro)* evolutionary design methods drawn from various bioengineering, digital and robotic ALife applications, while addressing highlighted directed evolution deficiencies.

## 1 Introduction

Artificial Life (ALife) is primarily defined as the study and creation of systems that exhibit life-like properties, with the objective of understanding the fundamental principles of living systems, such as self-organization, adaptation, and reproduction, through synthetic means (Bedau et al., 2007). However, there is no universally accepted definition of ALife, largely due to its interdisciplinary nature, which spans biology, computer science, and robotics (Heudin, 2006). Langton (1986) introduced the term ALife, describing it as the investigation of possible forms of life beyond those observed in nature (Langton, 1986), asserting that ALife seeks to understand life by constructing it, whether in software (soft ALife), hardware (hard ALife), or biochemical systems (wet alife) (Bedau, 2003). In this review, we adopt the definition of ALife by Langton (1986).

Synthetic biology is a bioengineering approach that uses engineering principles in biology, focusing on designing, building, and programming components such as DNA circuits, metabolic pathways, and RNA-based devices like ribozymes (Peng et al., 2021). It extends beyond living cells to cell-free platforms and nucleic acid–based molecular circuits (Pardee et al., 2014). Standardized biological parts, computational design, and rapid DNA synthesis enables the creation of new or enhanced biological functions for use in medicine, biotechnology, and environmental applications (Khalil and Collins, 2010; Marris and Calvert, 2020), just to mention a few.

Integrating ALife and Synthetic Biology presents a profound synthesis of interdisciplinary approaches that combine computational, biological, and engineering perspectives (Madhavan and Mustafa, 2023). Coupled together, these fields can bridge the gap between simulation and synthesis, creating a unified framework for investigating how life might arise, evolve, and be engineered across digital and physical domains and providing the foundation for hybrid, evolvable systems capable of purposeful function (National Research Council, 2014).

### 1.1 Foundations: ALife and synthetic biology

ALife has provided a platform for the evolution of self-replicating computer programs capable of mutating, evolving, and adapting in simulated environments (digital organisms) (Aguilar et al., 2014), supporting the mantra of *“life as it could be”* in *in silico* (simulation) systems as envisioned by Langton (1986) (Bedau et al., 2007). The mantra of *“life as it could be”* defines the full design and understanding of living systems, not just exclusive to nature, but abstracted from any specific medium and in contrast to biology’s study of *“life as we know it to be”* on earth (Dorin and Stepney, 2024). An *in vitro* (physical) system counterpart to *ALife* is synthetic biology (Brooks and Alper, 2021), an engineering-driven approach to biology with a special focus on designing and constructing new biological parts, devices, and systems, or redesigning existing natural biological systems for useful purpose (Heinemann and Panke, 2006). Synthetic biology aims to assemble modular biological building blocks with defined functions and interfaces (standardized biological parts) such as promoters, ribosome and binding sites that are well-defined, reusable, characterized, and programmable (Katz et al., 2018) into problem-solving synthetic (ALife) organisms (Endy, 2005), similarly addressing the ALife mantra of *“life as it could be”* (Forbes, 2000). Both ALife and synthetic biology provide experimental platforms for studying the fundamental biological principles of life, such as heritable information, variation and selection leading to evolution, energy-driven self-maintenance (metabolism) (Gánti, 2003), and the emergence of complex systems and evolution (de Vladar et al., 2017). This demonstrates the potential of ALife in the digital and physical worlds and are thus inexorably related. For example, various types of ALife existing *in silico* are artificially evolved computational analogues of living systems, exhibiting behaviors adapted to compete for resources (such as computer processor cycles) within a simulation (Adami, 2006). Current biotechnological applications enabled by synthetic biology are primarily limited to *in vitro* (controlled laboratory environments) and, to a lesser extent, *in vivo* (living organisms) contexts (Slomovic et al., 2015). However, future applications are expected to address global humanitarian challenges (Brooks and Alper, 2021), including improvements in health, food and water security, sustainable energy production, and environmental restoration (Pachauri et al., 2009). *On-demand* bioengineering, which involves rapid and precise design, construction, and deployment of biological systems and materials as needed, offers significant potential for applications such as vaccines (Hart and Ferguson, 2019), regenerative medicines (Peng et al., 2023), novel biomaterials (Szymanski et al., 2023), and sustainable products such as biofuels and plant-based foods (Voigt, 2020). Natural processes often employ regulatory architectures similar to those found in control, electrical, and robotic engineering; this similarity has motivated the application of control-theoretic methods for designing and implementing synthetic biological systems (Steel et al., 2017). Foundational technologies, including engineered gene regulatory networks, provide essential design frameworks for these advancements (Baier et al., 2023). Furthermore, synthetic biology-driven platforms have been proposed as foundational elements for Mars mission architectures (Nangle et al., 2020), where biomanufacturing of food, materials, therapeutics, and waste reclamation could support a self-sustaining Martian infrastructure (Santomartino et al., 2023). Although on-demand bioengineering is partially achievable today, with the ability to design and produce simple biological components rapidly, a significant gap remains in achieving robust, adaptable, and safe deployment (Brooks and Alper, 2021). Key capabilities, such as automated DNA synthesis, cell-free expression, and biofoundries, contribute to the feasibility of on-demand bioengineering; however, challenges persist in terms of predictive reliability, scalability, and containment (Brookwell et al., 2021). ALife addresses these challenges by enabling *in silico* evolution and digital-twin stress testing, thereby improving the transition from design to deployment (Hale and Stanney, 2014).

### 1.2 Challenges in deploying artificial life

There are two key problems that confound the synthesis and deployment of ALife for real-world applications. First, current bioengineering platforms (Grozinger et al., 2019) are mainly intentionally limited to function within controlled laboratory (*in vitro*) environments, serving as a critical bio-safety measure to prevent unintended environmental release or ecological impact (Gao et al., 2025). However, in some instances, this limitation is unintentional rather than caused by technical constraints, specifically the inability to accurately translate *in vitro* functioning systems into robust, self-sustaining entities capable of reliable performance in complex, real-world settings (Hitchins, 2008). This persistent gap between laboratory success and operational viability outside the laboratory remains a significant barrier to the development of deployable artificial life systems (Brooks and Alper, 2021). Second, there is no gold-standard method to direct the evolutionary design of organisms for optimal adaptation to a given task environment (application) (Wang et al., 2021) because designing a globally optimal organism requires balancing trade-offs between multiple, often competing, objectives, such as efficiency, robustness, and adaptability, rather than optimizing a single property (Vincent, 2017). Consequently, the synthesis of problem-solving ALife depends on predicting evolutionary trajectories, which in turn requires sufficient knowledge of the organism’s underlying fitness landscape (Wortel et al., 2023). A fitness landscape is a conceptual framework that can be visualized as a multidimensional surface, where each genotype occupies a position in sequence space and its corresponding fitness value determines the height at that point (Poelwijk et al., 2007). At the molecular level, the sequence space is the combinatorial space of all possible nucleotide or amino acid sequences (Povolotskaya and Kondrashov, 2010). In this review, we specifically refer to proteins and nucleic acids, where the notion of sequence space is well defined. Fitness is a flexible and context-dependent measure of performance according to a specific function or goal, such as catalytic activity in ribozymes (Schoenle et al., 2018) and the fitness function is the specific rule or calculation used to assign a quantitative score that measures the goodness of any possible solution for a given problem (Wang et al., 2021). Fitness landscapes have been demonstrated as critical to optimization in various applications, beyond synthetic biology (Yi and Dean, 2019), including ecology (Sanchez-Gorostiaga et al., 2019) and biomedicine (Nichol et al., 2015). Mapping fitness landscapes has been demonstrated as notoriously difficult (Poelwijk et al., 2007), such that predicting and controlling the evolutionary trajectories of bioengineered agents remains one of the significant obstacle to synthetic (biological) ALife reaching its full potential (Lässig et al., 2017). Although understanding fitness landscapes is not the sole limitation in constructing synthetic life, it illustrates significant methodological and conceptual challenges (Brun-Usan et al., 2022). These challenges restrict the integration of molecular, cellular, and system-level processes into unified, evolving artificial systems (Rothschild et al., 2024). This article thus focuses on the methodological deficiencies in mapping fitness landscapes and directed evolution as core mechanisms driving the evolutionary design of synthetic ALife. Artificial evolutionary design, inspired by natural evolution as the core proponent of biological design, diversity and adaptivity, has been a constant source of novel and innovative solutions to a vast array of design and optimization problems (Eiben and Smith, 2015a).

### 1.3 A hybrid synthetic perspective on artificial life

To further the state-of-the-art of ALife evolutionary design methodologies, we propose future research guidelines for novel Hybrid ALife methodologies that combine *in silico* (computational) and *in vitro* (biological) design of synthetic problem-solving ALife. Various definitions of Hybrid ALife have been proposed (Baltieri et al., 2023), but such work does not account for experimental (*in vitro*) evaluation of biologically synthesized ALife agents. Historically, ALife design methodologies have been grounded in evolutionary design on digital (*in silico*) (Sims, 1994; Yaeger, 2009; Auerbach and Bongard, 2014; Joachimczak et al., 2016; Kriegman et al., 2018) or physical (robotic) (Lipson and Pollack, 2000; Brodbeck et al., 2015; Jelisavcic et al., 2017; Hale et al., 2019) experimental platforms with relatively little work in experimental validation of synthetic ALife (Blackiston et al., 2021; Bongard and Levin, 2021). If synthetic ALife is to be designed *on-demand* for various real-world applications (Brooks and Alper, 2021), then we will require something akin to a Hybrid-ALife tool-kit, integrating *in silico* and *in vitro* tools to enable directed evolutionary design of synthetic ALife. Specifically, just as synthetic biologists construct (bioengineer) biological *devices* by assembling standardized biological parts such as (*BioBrick* parts (iGEM Foundation, 2025)) into problem-solving biological *systems* (Stock and Gorochowski, 2024), we advocate extending current ALife research into a hybrid (computational and biological) evolutionary design methodologies that enable ALife researchers to automate the design of synthetic problem-solving ALife. BioBrick parts are standardized DNA sequences with specific functions that, when assembled, implement more complex behaviors (Shetty et al., 2008). Throughout this article, we refer to individual BioBrick parts as functional sequences, and assemblies of BioBrick parts as complex synthetic biological devices, which we treat as *agents*.

Adaptive walks play a critical role in the design of ALife, they are a stepwise evolutionary process that begins with an initial sequence, often referred to as the wild type or reference sequence and progresses toward sequences with higher fitness (Sarkisyan et al., 2016). From the starting sequence, adaptive walks explore the fitness landscape, a conceptual representation that maps genotypes to their corresponding fitness values (Greenbury et al., 2022). They sequentially accept beneficial mutations and move toward regions of higher fitness within the landscape, demonstrating the importance of the topography of the fitness landscape (De Visser and Krug, 2014). The fitness landscape topography is characterized by a pattern of regions representing high-fitness genotypes (peaks), low-fitness regions (valleys), and neutral zones (plateaus) and this pattern determines how easily evolution can proceed towards optimal solutions (Weinreich et al., 2018). Consequently, this strongly affects how readily organisms can adapt and how robust they are to mutations (Smerlak, 2021). To explain robustness and adaptation, theories of neutral (molecular changes that are neutral and have no effect on fitness) (Kimura, 1989) and nearly neutral (molecular changes have tiny-effect changes influenced by population size) (Ohta, 2002) evolution suggest that many genetic changes have little or no immediate effect on fitness. The presence of neutrality (vast majority of evolutionary changes at the molecular level are caused by random genetic drift acting on mutations that have no effect on the fitness of an organism) promotes broad search of the fitness landscape (exploration), allowing populations to move freely across the landscape and potentially discover new adaptive peaks, thereby fostering the emergence of new traits (evolutionary innovation) and the capacity to respond to changing environments (adaptability) (Gray et al., 2010; Draghi and Plotkin, 2011). Evidence supporting these ideas comes from experiments in computer-simulated evolutionary systems (digital evolution) (Franklin et al., 2019), as well as studies of RNA structures (Hayden et al., 2011), proteins (Podgornaia and Laub, 2015), and gene-regulatory networks (Baier et al., 2023). Despite these insights, there is no standardized method for directly reconstructing or inferring fitness landscapes from biological data, nor is there a formal mathematical expression of fitness applicable across systems (Wang D. et al., 2024). The challenge is heightened by the extremely high dimensionality of these landscapes, the complex epistatic interactions between genes (when the effect of one mutation depends on the presence of others), and the constantly changing nature of these interactions due to ongoing mutation and natural selection (De Visser and Krug, 2014). Moreover, limited empirical data on how mutations and epistasis reshape fitness landscapes continue to hinder accurate mapping and predictive models for directed evolution (Wang et al., 2021).

Given this, we first review directed evolution as it applies to bioengineering and open issues with directed evolution to be addressed if *on-demand* evolution of synthetic ALife is to become a reality. Second, we review fitness landscape mapping as it relates to directed evolution in bioengineering, specifically, the disconnect between empirical fitness landscapes mapped *in silico* and evolving fitness landscapes in nature (Fragata et al., 2019). Third, we propose future work guidelines for a Hybrid ALife methodology as a potential way forward for on-demand design (directed evolution) of synthetic ALife problem-solving agents. Throughout this review, we focus on *in vitro*, as opposed to *in vivo* methods as the biological substrate for validating directed evolution methods. This is because directed evolution methods operating in controlled *in vitro* (laboratory) environments enable fine-grained control of genetic sequence mutation and validation. Also there is no generalized method to assess generated sequence fitness *in vivo*, meaning the exact impact of sequences evolved *in vivo* across various biological substrates is difficult to ascertain (Vidal et al., 2023). Hence, we limit discussion in this review to the evolutionary design of synthetic ALife agents with potential applications limited to controlled and structured environments such as laboratories and factories. An important final caveat is that we purposely do not refer to, and thus review and critique, chemically synthesized biological ALife or *wetware* as it is popularly known in the ALife community (Aguilar et al., 2014). We instead defer to current bioengineering (synthetic biology) research, which addresses many of the same fundamental questions as wetware research. This is especially the case since this article’s purview is limited to evolutionary design for the purpose of synthesizing problem solving (ALife) agents for specific applications, which extends the scope of many bioengineering efforts (Voigt, 2020).

## 2 Directed evolution

Directed evolution (DE) is a closed loop evolutionary optimization that mimics the process of natural evolution, but on a shorter timescale, where a the specific rule or calculation used to assign a quantitative score that measures the goodness of any possible solution for a given problem) objectifies (fitness function) a specific target, and thus individuals are selected for improvement based on measured performance according to a specific function or goal, such as catalytic activity, binding affinity, growth rate and substrate specificity (fitness) (Knowles, 2010). Recent advances in machine learning have been applied to optimise directed evolution, leading to greater efficiency and predictive accuracy (James et al., 2024). However, most existing methodologies rely on DNA sequencing and synthesis, rendering them resource-intensive and less compatible with emerging *in vivo* mutagenesis techniques (James et al., 2024). In response to these limitations, James et al. (2024) explored the *GB1* and *TrpB* empirical landscapes without sequencing information and demonstrated up to *19-fold* and *7-fold* increases, respectively, in the probability of reaching the global fitness peak. In the evolutionary design of ALife, directed evolution has produced a diverse range of simulated and robotic organisms, demonstrating the artificial evolution of various forms of *“life as it could be”* (Aguilar et al., 2014). In bioengineering, directed evolution enables the rapid selection of tiny building blocks of life (biomolecules) with properties suitable for applications such as protein engineering (Packer and Liu, 2015), isolating new enzyme variants with improved function (Currin et al., 2021), generating novel bio-catalysts (Coelho et al., 2013), and engineering genetic parts and synthetic gene circuits (Wang et al., 2021) as the biological components of various bioengineering applications (Cubillos-Ruiz et al., 2021). Directed evolution is becoming increasingly important as an experimental tool to bioengineer holistic genetic constructs containing multiple biological components (Vidal et al., 2023) (synthetic ALife). Since the first *in vitro* evolution experiments (Mills and Spiegelman, 1967), many techniques have been developed to address the two key steps of directed evolution: genetic diversification (library generation), and isolating genetic variants of interest (Vidal et al., 2023).

Directed evolution modalities can be classified into three categories: traditional directed evolution (Cheng et al., 2015), growth-coupled directed evolution (Chen et al., 2022), and AI-assisted directed evolution (Yang et al., 2025). Traditional and growth-coupled approaches rely primarily on experimental diversification and selection, whereas AI-assisted directed evolution augments or partially replaces empirical screening with model-guided design. Complementary strengths between these frameworks exist as traditional and growth-coupled methods offer robust, biology-native selection regimes but can be experimentally intensive, while AI-assisted DE can accelerate convergence and explore sequence space more efficiently, at the cost of increased dependence on data quality and modeling assumptions. Table 1 presents a comparison of the three modalities.

**TABLE 1:** Comparison of traditional DE, growth-coupled DE, and AI-assisted DE.

Traditional DE	Growth-coupled DE	AI-assisted DE
Phenotypic activity is measured directly through biochemical or cell-based assays	Performance is inferred from cellular fitness or growth advantage under selective conditions	Fitness or target property is predicted computationally and experimentally validated
Empirical and largely stochastic exploration of sequence space via random or semi-rational mutagenesis	Sequence exploration driven by endogenous or engineered selection pressures	Sequence space navigation guided by machine-learning models that prioritize promising variants
Throughput limited by assay capacity and manual screening	Highly scalable due to population-level growth selection	Scalability enhanced by computational filtering that reduces the experimental search space

### 2.1 Computational and experimental directed evolution

In bioengineering, directed evolution is formulated as an algorithmic process that mimics natural selection to improve biomolecules with desired properties (Yang et al., 2019). It involves generating genetic (DNA) diversity for a specific individual, expressing the genetically diversified DNA through transcription and translation systems, screening the resulting sequences, and then selecting specific mutant sequences for the next round (Wang et al., 2021). During transcription the information encoded in DNA is converted into a transient carrier of information (mRNA), while translation is the process by which the mRNA code is converted into an amino acid polymer by ribosomes (RNA catalysts called ribozymes) (Taylor, 2006; Cech, 2000; Engstrom and Pfleger, 2017). Similarly, in *in silico* (Sims, 1994; Auerbach and Bongard, 2014; Joachimczak et al., 2016; Kriegman et al., 2018) and robotic (Lipson and Pollack, 2000; Brodbeck et al., 2015; Jelisavcic et al., 2017; Hale et al., 2019) ALife systems, evolutionary design is governed by evolutionary algorithms, which simulate the same principles of variation and selection (Bartz-Beielstein et al., 2014). Initialized with a population of randomly generated solutions, the algorithm enters a loop where it evaluates each generation of solutions, assigns a fitness (or solution quality), and applies variation operators (mutation and crossover) to the fittest subset to create the next-generation (Eiben and Smith, 2015a). The solution quality of directed evolution is driven by the selection (fitness function) and exploration of the search space (fitness landscape), which are determined by these variation operators (Wang et al., 2021). Genetic diversity methods in computational and bioengineering models are further discussed in Sections 3.1, 3.2. The importance and challenges of fitness landscape mapping are explained in detail in Section 5. Parallels between experimental and computational approaches to generating genetic diversity are summarized in Table 2.

**TABLE 2:** Parallels in experimental and computational strategies for genetic diversity generation.

Bioengineering strategy	Computational analogue	Shared objective
Mutagenesis	Mutation operators	Introduce small-scale genotypic variation
Recombination	Crossover	Combine solutions to explore new regions
Adaptive random walks	Mutation-only evolutionary search	Incremental exploration of local fitness neighborhoods
CRISPR/guided edits	Constraint-aware variation	Targeted sampling of promising subspaces
Error-prone DNA synthesis	Quality-diversity generation	Broad, structured coverage of genotype space

### 2.2 Fitness landscapes and adaptive dynamics

Selection refers to the process by which organisms possessing advantageous traits are more likely to survive and reproduce, thereby transmitting these traits to their offspring (Bell, 2008). This process serves as the primary mechanism driving the evolution of diverse forms, functions, complexity, and behaviors observed in ALife (Gershenson, 2023), where bioengineering relies upon a natural selection approximation (fitness) (Barton, 2022). Specifically, in the *in vitro* evolutionary environments of bioengineering laboratories, sequence fitness is equated with function performance such as relative catalytic rates of enzymes (Rotrattanadumrong and Yokobayashi, 2024) or observed fluorescence of protein genotypes (Sarkisyan et al., 2016), while ALife evolution *in silico* equates agent fitness with solution quality, represented as a numerical value. In both cases, a conceptual representation that maps genotypes to their corresponding fitness values (fitness landscape) (Hartl, 2014) is assumed to underlie evolutionary adaptation of individual agents, where the heritable differences in the genetic material and its arrangement among the population (genetic variation) driven by mechanisms such as mutation and recombination are coupled with selection, enabling the propagation of higher fitness individuals over evolutionary time (Mitchell-Olds et al., 2007).

An ongoing challenge in the evolutionary design of problem-solving ALife, for example, evolved robotic agents (Lipson and Pollack, 2000; Brodbeck et al., 2015; Jelisavcic et al., 2017; Hale et al., 2019), is premature convergence to sub-optimal solutions (for example, robotic forms and functions). Similarly, directed evolution of sequence design for various bioengineering applications (Currin et al., 2021) also suffer from convergence to sub-optimal solutions. This is typically a result of poor or highly localized search of the fitness landscape underlying the evolution of a genetic individual (sequence) (Firnberg et al., 2014). Recent studies of empirically mapped fitness landscapes demonstrate that epistatic interactions, particularly reciprocal sign epistasis, can create barriers that render certain fitness peaks inaccessible from specific ancestral genotypes, thereby constraining global optimization even in computationally or experimentally evolved systems (De Visser and Krug, 2014; Nishikawa et al., 2021; Poelwijk et al., 2011; Weinreich et al., 2005).

### 2.3 Exploration and exploitation in evolutionary search

As in the evolutionary design of ALife agents with desired forms and functions, bioengineering applications must similarly balance locally optimizing variants around known high-fitness sequences (exploitation) versus searching widely in sequence space for novel functional solutions (exploration) in the evolutionary search (Crepinsek et al., 2013) of the fitness landscape. For example, via enabling sufficient exploration of new solutions (via genetic variation operators) while concurrently exploiting (via selection operators) already discovered solutions (Wang et al., 2021). Practically, this means maintaining sufficient genetic diversity in solutions so as enough effective (high fitness) solutions can be screened, synthesized, tested and validated (Currin et al., 2021). Achieving an exploration (broad search) versus exploitation (local optimization) balance is especially prevalent in bioengineering given the expense of experimentally verifying solutions (*in vitro*), and added risks of wasting time and funds on synthesizing sub-optimal solutions. Realistically, as many sequences as practically possible should be screened and evaluated, meaning that any evolutionary search process enacted by bioengineering applications are orders of magnitude more expensive than their *in silico* evolutionary design counterparts. This expense is calculated in terms of computational run-time, the fiscal cost of producing synthetic sequences and the time taking for testing synthesized sequences (Reetz et al., 2008). This accentuates the critical importance of balancing exploration (broad search) versus exploitation (local optimization) in the intractable search space of genetic sequences (Currin et al., 2015). Thus, if hybrid ALife systems are to be efficient (minimizing experiment resources) in the evolutionary design of effective (problem-solving) ALife agents, then such an evolutionary design processes must suitably balance exploitation (optimization) versus exploration (broad search) of the fitness landscape underlying ALife agent design. An open issue that continues to pervade evolutionary design in ALife and bioengineering applications alike is the limited discovery of new genotypes or phenotypes that gives the population achieve higher fitness or new adaptive fitness function (novel solutions), where such limitations are determined by the capabilities of genetic variation operators, generating feasible novel solutions that can be selected and evaluated by the evolutionary design process.

## 3 The directed evolution problem: generating genetic diversity

There has been considerable progress in applying directed evolution to synthesis of fundamental biological components, for example, enabling *de novo* designs of functional proteins with new topologies, assemblies, binders, catalysts and materials (Cao et al., 2022). However, current macromolecule designs produced by directed evolution methods are still far from the complexity and variety of those found in nature (Ingraham et al., 2023). This is because such directed evolution methods rely on iterative search and incremental sampling of highly rugged and complex fitness landscapes (Wang et al., 2021). Given the limited sampling possibility in laboratory timescales, directed evolution typically explores only a small fraction of sequence space (Currin et al., 2015). While natural evolution itself is not necessarily globally optimal and is shaped by historical contingencies and co-evolutionary constraints, it has nonetheless explored vast regions of sequence space over billions of years, resulting in a high diversity of effective natural solutions (Koskella et al., 2017). Consequently, directed evolution may often rediscover or modestly improve upon natural functionalities rather than consistently surpass them (Turner, 2009). An overview of algorithmic applications in directed evolution is provided in Table 3.

**TABLE 3:** Algorithms and their applications in directed evolution.

Algorithm family	Core mechanism	Typical application
Evolutionary algorithms	Mutation, recombination, selection operators	Simulated and robotic ALife, evolutionary design
Adaptive random walks	Local mutation-based exploration	Protein and enzyme optimization
Hybrid diversity maintenance methods	Balance exploration and exploitation via novelty or hybrid search	Synthetic ALife and bioengineering design
Predictive machine learning	Supervised learning from sequence–function data	Protein and RNA function prediction
Generative machine learning	Latent-space sampling (e.g., VAEs, LLMs)	Novel sequence generation for proteins and RNAs
Integrated ML–evolution pipelines	Iterative data-driven selection and synthesis cycles	Automated directed evolution workflows

### 3.1 Genetic diversity in evolutionary algorithms

In evolutionary design (computation) algorithms, as the fitness landscape dimensionality increases, the effectiveness of evolutionary search degrades, leading to sub-optimal solution convergence. Addressing such sub-optimal algorithmic convergence requires solution diversity maintenance. Diversity maintenance methods either maintain genotype (solution encodings on the fitness landscape), or phenotype diversity (solution diversity in the application space) (Eiben and Smith, 2015a). Genotypic and phenotypic diversity maintenance are well explored topics in evolutionary ALife design (Cully and Demiris, 2018; Miras and Ferrante, 2020; Nordmoen et al., 2021; Mkhatshwa and Nitschke, 2024). Some key strategies for maintaining and increasing genetic diversity include *niching*, *fitness sharing*, *crowding*, and *multi-population models* (Sareni and Krahenbuhl, 1998). Niching encourages subsets of solutions to specialise in distinct regions of the problem space, promoting the formation of diverse subpopulations (Li et al., 2016). Fitness sharing reduces the effective fitness of an individual by dividing its raw fitness by a factor representing the density of similar individuals nearby (Della et al., 2004). Crowding ensures that a new offspring replaces only the most similar individual in the current population (Sng et al., 2017). Finally, multi-population models prevent premature convergence by maintaining separate evolutionary lines that are less likely to become trapped in the same local optimum (Li et al., 2015). Together, these methods encourage broader exploration of the solution space. Phenotype diversity is essential for exploring a wider range of solutions and is closely linked with quality diversity (Mouret and Doncieux, 2012). Quality Diversity (QD) algorithms aim to generate not just high-performing but also varied solutions (Pugh et al., 2016). Popular examples include *Novelty Search* (Pugh et al., 2016), which rewards the discovery of new behaviors rather than optimizing performance directly, and *MAP-Elites*, which organizes and preserves diverse high-performing solutions across different behavioral dimensions (Mouret and Clune, 2015). In phenotype diversity maintenance the search for phenotypically diverse solutions replaces the fitness function, meaning an encoded solution (genotype) is more likely to be selected for evolutionary variation and propagation if its phenotype (behavior) is sufficiently different and fit compared to solutions discovered thus far (Pugh et al., 2016). Phenotypic diversity maintenance has been successfully applied to boost the quality of evolved ALife organism designs (Cully and Demiris, 2018; Miras and Ferrante, 2020; Nordmoen et al., 2021; Mkhatshwa and Nitschke, 2024). In either case, solution diversity is derived from mutation of an individual’s genotype.

### 3.2 Genetic diversity in bioengineering

Mutation serves as a fundamental source of genetic diversity, and facilitates the enhancement of a population’s evolvability and fitness (Wagner, 2023). Genetic stability in engineered cell populations remains a significant challenge in synthetic biology, as synthetic constructs impose burdens on host cells and promote mutations that can eliminate engineered functions (Sechkar and Steel, 2025). Previous mutation-control strategies have typically relied on gene-specific designs, which limit their general applicability (Sechkar and Steel, 2025). Sechkar and Steel (Sechkar and Steel, 2025) proposed a universal biomolecular controller that suppresses mutant growth regardless of circuit identity, demonstrating enhanced performance and adaptability through resource-aware modeling and simulation. A prevalent example is of mutations in the SARS-CoV-2 genotype leading to changes in the protein structure (genetic diversity) resulting in an increase in the infectiousness, fitness, and virulence of the SARSCoV-2 virus (DeGrace et al., 2022). In bioengineering, approaches for generating genetic diversity include adaptive random walks (Rotrattanadumrong and Yokobayashi, 2024) of the conceptual representation that maps genotypes to their corresponding fitness values (fitness landscape), reshuffling of hereditary information by exchanging segments between related sequences, creating new combinations (*recombination*) either through DNA shuffling (Stemmer, 1994), and unpredictable changes throughout a genetic sequence to create a wide variety of variants (random *mutagenesis*). The latter is done *in vitro* using specially designed alterations in synthetically derived DNA (Morrison et al., 2020), or *in vivo* via genotype engineering tools such as CRISPR (Naseri and Koffas, 2020). Such genetic diversity generation methods enable the sampling and testing of a broader range of genetically diverse sequences, which is tantamount to evolutionary computation diversity maintenance methods such as quality-diversity (Pugh et al., 2016), where both approaches are designed to improve fitness landscape exploration and thus solution quality in directed evolution (Wang et al., 2021).

Adaptive random walks which are a stepwise evolutionary process that normally start with the wild type or reference sequence, and progresses toward sequences with higher fitness are the simplest approach to fitness landscape exploration (broad search) and thus discovery of genetically diverse solutions (Emmeche, 1996). Such random walks explore the landscape via enabling a given sequence to move to a neighboring sequence, for example, via a single mutation, with a probability proportional to the fitness of that sequence (Papkou et al., 2023). Such an adaptive walk process models a low mutation rate where mutants of a given sequence have only a single mutation, though higher mutation rates enable greater exploration of the fitness landscape (Saha et al., 2024). Adaptive random walks are analogous to evolutionary algorithms using mutation only (Eiben and Smith, 2015a), and have been successfully applied to explore the fitness landscapes of synthetic *Escherichia coli* (Santo et al., 2023) and RNA sequences (Wagner, 2023). Another popular exploration (diversity generation) method is recombination (DNA shuffling), where homologous sequences are fragmented and reassembled (Stemmer, 1994). DNA shuffling has been applied as a means of to assist directed evolution of proteins, enzymes, and metabolites (Zhang et al., 2019), for example, using the *Synthetic Chromosome Rearrangement and Modification by LoxP-mediated Evolution* (SCRaMbLE) method (Ma et al., 2019), for *in vitro* genotype rearrangement. Similarly, random mutagenesis methods explore the fitness landscape of a genetic sequence with precise, controlled mutations of the given sequence (thus generating genetic diversity), using, for example, targeted mutagenesis and *error-prone polymerase chain reaction* (epPCR) (Wong et al., 2006).

Alternate methods for generating genetic diversity *in vitro* have been proposed in the form of DNA synthesis methods (Wang and Vasquez, 2023). For example, Wang B. et al. (2024) proposed a directed evolution approach for *in vitro* genetic diversification in artificial DNA synthesis, a foundational technology in synthetic biology based on the assembly of synthetic oligonucleotides into double-stranded DNA. One method is *error-prone Artificial DNA Synthesis* (epADS), where base errors generated during the chemical synthesis of oligonucleotides are treated as random mutations, enabling epADS to introduce diverse mutation types (including base substitutions and indels) for *in vitro* genetic diversification of synthetic DNA. Applications studied included genetic diversification of synthetic circuits, and microbial cells demonstrating various levels of phenotypic modification (Wang B. et al., 2024). Furthermore, these computational design methods support modularity in synthetic biology, allowing the generation of standardized biological parts and their assembly using widely adopted synthetic biology assembly workflows, ensuring high interoperability, scalability, and facilitate practical adoption.

### 3.3 From evolutionary limits to computational design

Although such fitness landscape exploration (genetic diversity generation) methods have been successfully demonstrated for various *in vitro* applications (Müller et al., 2005), the complex, rugged, high-dimensional topography of fitness landscapes presents significant challenges for their broader use (De Visser and Krug, 2014). Such exploration methods are still deficient for eliciting broad genetic diversity maintenance and thus an extensive fitness landscapes exploration (Müller et al., 2005). This failure largely results from the inherently high dimensionality and ruggedness of fitness landscapes, where local optimization and limited sampling prevent exhaustive exploration, while statistical or smart library designs further narrow the search to preselected sequence subsets (Van Cleve et al., 2015). Consequently, poor exploration of fitness landscapes significantly limits the quality of solutions discovered by directed evolution. Various experimental improvements to synthetic biology methods have been proposed to improve the efficacy of exploration, and they are highly compatible with synthetic biology because their modular structure aligns effectively with established assembly methodologies. These experiments include creating *smart* libraries (Tang et al., 2016) of reduced diversity sequences to restrict the search space to statistically designed variants, thus improving process efficiency by lowering the portion of mutated sequences (Currin et al., 2021). Prevalent examples include: *CodonGenie* (Swainston et al., 2017), *ANT* (Engqvist and Nielsen, 2015), *SwiftLib* (Jacobs et al., 2015), *DYNAMCC* (Halweg-Edwards et al., 2016), *DC-Analyzer* and *MDC-Analyzer* (Wang et al., 2015). However, while such digital libraries reduce the experimental workload via reducing the screening duplicate or unwanted variants, such approaches assume that the selected variant sequences (stored in smart libraries) are already near optimal for the tested conditions; however, their effectiveness may still vary depending on the specific genetic and environmental contexts in which the genetic components are implemented. Such *in silico* tools have demonstrably enhanced the entire experimental process, for example, reducing manual intervention in experiment setup via integrating assembly designs with laboratory automation scripts to increases throughput and accuracy (Carbonell et al., 2016).

However, optimal use of artificial evolution as the primary design mechanism in bioengineering applications remains elusive, given that directed evolution is often confounded by erroneous assumptions that evolutionary trajectories always follow adaptive walks (Williams, 1996), and unanswered questions about how evolutionary trajectories can span fitness landscape valleys (Steinberg and Ostermeier, 2016). To mitigate such open issues recent bioengineering applications have gone beyond the evolutionary operators of mutation and recombination, with the *in vitro* synthesis of new sequences, optimized *in silico* with predictive and generative machine learning (Wittmann BJ. et al., 2021). In such approaches, high fitness sequences are selected by the experimenter as a starting point for further optimization, synthesis, and testing in the design-build-test cycle of bioengineering (Carbonell et al., 2016).

## 4 Directed evolution: what now and what next?

Evolutionary design, employing various recombination and mutation variation operators (Eiben and Smith, 2015a), has been successfully applied to explore fitness landscapes and direct the evolution of *in silico* ALife for decades (Wilke et al., 2001). Similarly, various analogues of artificial evolution operators (Eiben and Smith, 2015a), such as random *mutagenesis* (Copp et al., 2014) and DNA shuffling (Stemmer, 1994), have been applied for fitness landscape exploration and directed evolution in various bioengineering applications (Currin et al., 2021). The simplest artificial evolution analogue in directed evolution for bioengineering is the adaptive random walk (Papkou et al., 2023), where the fitness landscape is explored via generating and evaluating the fitness of mutant sequences from a given high fitness *wild-type* sequence (Rotrattanadumrong and Yokobayashi, 2024). Though, such adaptive random walks do not currently employ equivalents of the myriad of selection operators (Eiben and Smith, 2015a) available to the directed evolution of simulated ALife (Aguilar et al., 2014). Rather, selection operators in the directed evolution of bioengineering applications are replaced with experimental selection using specially designed mutant screening (biochemical assay) processes (Duong-Trung et al., 2023).

### 4.1 The combinatorial constraint

Regardless of random walk functionality, few fitness landscapes can be completely (exhaustively) mapped (Rowe et al., 2010) as the underlying sequence space is astronomically large (Rowe et al., 2010), though partial mappings have provided valuable insights to landscape topography (ruggedness) for specific sequence types such as proteins (Currin et al., 2015). For instance, a protein of 100 amino acids could, in principle, adopt a vast number of possible sequences, far exceeding the number of atoms in the observable universe (Koonin et al., 2002). Similarly, a 100-nucleotide RNA has potential variants, and even considering only single and double mutants of a 300-residue protein already involves over 16 million sequences (Keller et al., 2018). This combinatorial explosion renders exhaustive mapping infeasible; nevertheless, partial mappings have provided valuable insights into the topography of fitness landscapes for specific sequence types such as proteins (Kondrashov and Kondrashov, 2015). Hence, just as the fitness landscape metaphor has guided the directed evolution of simulated and robotic forms of ALife (Lipson and Pollack, 2000; Joachimczak et al., 2016; Jelisavcic et al., 2017; Hale et al., 2019), fitness landscape mappings provide valuable observations that assist in guiding directed evolution methods and the efficacy of sequence design in bioengineering more generally (Castle et al., 2024). The problem of balancing exploration versus exploitation in any directed evolution method continues to confound simulated, robotic and synthetic ALife alike, though various genotype and phenotype diversity maintenance methods (Pugh et al., 2016), including non-objective based (novelty) search (Lehman and Stanley, 2011), and hybrids of non-objective and objective (fitness function) based evolutionary search (Nitschke and Didi, 2017), have demonstrated improved designs of various forms of ALife (Cully and Demiris, 2018; Mkhatshwa and Nitschke, 2024), including unexpected, non-intuitive designs (Lehman et al., 2020). Such diversity maintenance methods have also been proposed as means to boost fitness landscape exploration and thereby continually discover novel synthetic ALife designs for bioengineering applications (Stock and Gorochowski, 2024).

### 4.2 Continuous evolution platforms

Rapid advances in continuous directed evolution have led to many new experimental platforms that closely combine *in vivo* diversification, automated selection, and real-time measurement. Among these, PACE, OrthoRep, EvolvR, and automated continuous-evolution systems are the most advanced for sustained, hands-free evolution of biomolecules. These platforms utilize various methods to connect genetic diversification to organism growth or phage propagation, thereby reducing manual work. They offer strong tools for closed-loop optimization through the build–test–select cycle with automation.

Phage-assisted continuous evolution (PACE) is considered the first broadly applicable system for fully continuous *in vivo* (in living organisms) directed evolution (Esvelt et al., 2011). Evolving genes are carried on filamentous phage (viruses that infect bacteria) that propagate through *E. coli* (a common laboratory bacterium) in a chemostat-like (continuous culture) setup, where phage infectivity, and thus replication rate, is directly tied to the activity of the biomolecule under selection (Arnold, 1998). This architecture enables numerous evolutionary rounds with minimal intervention, significantly accelerating and deepening evolutionary searches compared to batch (stepwise) methods (Arnold, 1998). PACE has been utilized to evolve diverse protein functions, including altered protease (enzyme) and DNA-binding specificities, and deep sequencing (analyzing DNA with high coverage) has revealed the underlying adaptation pathways (Miller et al., 2020). Together, these advances establish PACE as a mature, partially automated platform well-suited for integration with computational design and analysis.

OrthoRep is a yeast-based continuous evolution system that utilizes an orthogonal DNA polymerase plasmid pair to drive mutation rates ∼ 10^5^-fold above those of the yeast genome, while preserving genomic stability (Ravikumar et al., 2018). Genes placed on the orthogonal plasmid undergo continuous error-prone replication during routine culturing, enabling scalable evolution through simple serial (Ravikumar et al., 2014). OrthoRep has been used to evolve drug-resistant malarial DHFRs, probe large ortholog sequence spaces, and optimize enzymes for plant metabolic engineering, demonstrating deep, multi-lineage evolutionary searches with minimal handling (Rix et al., 2020). Overall, OrthoRep is a robust platform for continuous gene diversification and selection, supporting computational frameworks that propose targets, lineages, or selection strategies.

EvolvR is a CRISPR-guided (Cas protein and guide RNA-based), locus-specific (targeting exact DNA sites) continuous diversification system that can target either genomic (organismal DNA) or plasmid (circular DNA) sequences (Halperin et al., 2018). It utilizes a Cas9 nickase (a DNA-cutting protein that creates single-strand breaks) fused to an error-prone polymerase (a mutation-adding enzyme), with guide RNAs directing mutations to user-defined loci within a tunable window around the nick site (the location of the DNA cut) (Halperin et al., 2018). In this way, EvolvR generates focused mutational clouds (clusters of variants) during growth (Halperin et al., 2018). This localized diversification enables parallel evolution of multiple genetic elements and, importantly, can be re-targeted as design goals or inferred fitness landscapes (relationships between genetic changes and organismal function) change (Halperin et al., 2018). In subsequent work, EvolvR has been positioned within a broader class of CRISPR-based evolution tools (Abbott and Qi, 2018). This highlights its potential for closed-loop (iterative, feedback-driven) optimization of genetic parts, regulatory sequences, and enzymes when combined with automated assays and computational design.

Automated continuous-evolution (ACE) systems combine *in vivo* mutagenesis (mutation induction within living cells) with programmable hardware to enable hands-free, feedback-controlled evolution over long timescales (Zhong et al., 2020). The ACE platform links OrthoRep’s targeted hypermutation in yeast to the eVOLVER system, an array of independently controlled culture vessels equipped with automated dilution (automatic culture thinning), temperature regulation, and media switching (changing growth liquid), enabling software-defined adjustments of selection pressures (factors determining survival) and culture conditions (Heins et al., 2019). These setups have evolved enzymes with altered temperature optima (best activity temperatures) and catalytic properties over hundreds of generations with minimal intervention, demonstrating how continuous evolution, real-time monitoring, and programmable feedback can operate as a unified platform.

Most continuous evolution platforms optimize only phenotypes that are readily measurable, such as simplen *growth - infectivity* linked traits. As a result, these systems tend to fine-tune narrow functions rather than reveal complex or context-dependent behaviors. They also depend heavily on specific chassis organisms and fixed culture conditions, which can further constrain evolution to a particular cellular environment. This dependence reduces the portability of evolved variants and limits the exploration of diverse biological contexts. Additionally, the prevailing mutation modes and population dynamics promote dense local search rather than large evolutionary leaps, often causing systems to become trapped on local fitness peaks and overlook rare but innovative solutions. Collectively, these constraints enable rapid optimization within a defined niche but restrict these platform’s capacity to generate truly novel and broadly adaptable functions.

### 4.3 Machine learning as a catalyst for evolutionary discovery

Predictive and generative machine learning methods provide a complementary approach to boost genetic diversity and address the exploration versus exploitation problem in directed evolution via discovering new functional sequences (Brya et al., 2021), where combinatorial mutations generate greater genetic diversity, accelerating the discovery of novel traits and optimizing adaptive potential in evolving organisms (Schlötterer et al., 2015). However, genetic sequence information is sometimes poorly represented, since non-functional patterns and high levels of expression are often under-represented in training data (Fernandez-de Cossi et al., 2021), which limits exploration of the fitness landscape and reduces overall benefit of machine learning methods in directed evolution. However, machine learning can increase the accuracy and efficiency of directed evolution methods via generating new genetically diverse sequences and predicting the efficacy of such newly generated sequences, such as proteins (Notin et al., 2024) and enzymes (Landwehr et al., 2020). For example, *Large Language Models* have been applied to protein discovery (Ingraham et al., 2023), generated to user specifications (Lin Z. et al., 2023). A study by Biswas et al. (2018) used predictive machine learning to address issue of directed evolution in protein engineering, where evolved proteins often became trapped in local solution space maxima, and yielding only marginal improvements (Romero and Arnold, 2009). They encoded protein sequences as learned amino acid embeddings and used them to train Linear Regression, Feed-Forward Networks, and a novel Composite Residues model with the goal to predict log fluorescence via mean-squared error loss, and the models jointly learned embeddings and parameters through empirical tuning. Multiple train-dev-test splits were implemented to evaluate the model’s generalization. Performance was benchmarked using the mean squared error and false discovery rate, with an emphasis on robustness in identifying functional variants. Biswas et al. (2018) demonstrated via combining supervised machine learning, DNA synthesis, and high-throughput screening that data-driven machine learning can predict novel, functional protein variants, thus sufficiently exploring the fitness landscape, and aiding directed evolution in optimizing protein design. The proposed approach by Biswas et al. (2018) provides an efficient directed evolution strategy for exploring unseen regions of the fitness landscape, escaping local maxima, and increasing the likelihood of finding useful protein variants, which traditional methods can struggle to find. However, despite its potential to improve the speed and efficiency of protein engineering, this model depends on the sufficiency of high-quality training data and the model’s capability to generalize beyond known variants is limited. In the absence of such resources, model generalization beyond known variants is limited, and predictions may fail to accurately represent biological reality (Ching et al., 2018). Furthermore, as demonstrated in large language models applied to biological data, generative and predictive models may produce plausible yet incorrect outputs, underscoring the necessity for rigorous benchmarking and experimental validation prior to deployment in practical protein design applications (Zhang et al., 2025).

One prevalent example is the work of Ding et al. (2019) used generative machine learning to address open issues with a protein inference method, heuristic phylogeny reconstruction, including the method’s failure to capture high-order epistasis effects. Using both simulated and experimental data, Ding et al. (2019) demonstrated that latent space models, trained using *Variational Auto-Encoders* (VAEs) and information within multiple sequence alignments of protein families, can help capture phylogenetic relationships, boost exploration of protein fitness landscapes, and thus aid in predicting protein stability change upon single-site mutations. Variational autoencoders were trained on one-hot encoded multiple sequence alignment data. Each sequence was mapped to a Gaussian posterior in a low-dimensional latent space, and the decoder produced per-position amino acid probabilities. For training, they employed the reparameterization trick with stochastic variational inference and mini-batch optimization. Sequence weights derived from the multiple sequence alignment were incorporated during model training. Modeling the protein sequence space using *Variational Auto-Encoders* (VAEs) created a condensed search space, increasing fitness landscape exploration and the likelihood of finding beneficial mutations. However, the VAEs summarize complex protein data meaning resulting representations often lack biological interpretability where this lack of interpretability hinders an explainable design process in directed evolution.

Another emblematic is where Angenent-Mari et al. (Wang et al., 2020) applied predictive machine learning to address open problems with effective design and validation of RNA molecules in the bioengineering of RNA molecules with targeted biological functions (Pardee et al., 2016). Specifically, Angenent-Mari et al. (Wang et al., 2020) developed a high-throughput DNA synthesis, sequencing, and deep-learning pipeline to design and analyze a programmable RNA switch, achieving a ten-fold improvement in functional prediction compared to traditional thermodynamic and kinetic models. The *Deep Neural Networks* (DNNs) pipeline proposed by Angenent-Mari et al. (Wang et al., 2020), generated attention visualizations (VIS4Map), providing a way to visualize sequence parts that contribute to the success or failure of the RNA switches, enhancing understanding of the underlying functionality and identification of critical features or motifs that drive RNA functionality. However, the DNN pipeline is based on sequence-function relationships, meaning it only accounts for some of the complex interactions present in RNA molecules, leading to potential over-simplifications in predicted (designed) RNA sequences.

Hence, even though there has been some cross-fertilization between evolutionary design, machine learning, and the design-build-test processes of bioengineering, synthetic biology still lacks an equivalent to the autonomous, iterative evolutionary processes used in simulated and robotic ALife, where systems evolve new functions without explicit human-guided design (Lipson and Pollack, 2000; Aguilar et al., 2014; Joachimczak et al., 2016; Jelisavcic et al., 2017; Hale et al., 2019). Though, various bioengineering case studies (Biswas et al., 2018; Ding et al., 2019; Wang et al., 2020) highlight a deficiency in methods to generate genetic diversity (discovering new genetic sequences) and overcome the confounding exploration versus exploitation problem of directed evolution. We envisage future synthetic ALife design-build-test processes as encapsulating complements of predictive and generative machine learning (Wittmann BJ. et al., 2021), hybrid (objective and non-objective based) diversity maintenance operators and adaptive random walks using recombination and mutagenesis. *Design*, would thus be novel sequence discovery (generated genetic diversity) achieved via generative machine learning or evolutionary operators (recombination or mutagenesis). The probable efficacy (fitness) of discovered sequences could be gauged by data-driven predictive machine learning and experimentally *built* (*in vitro*), to *test* the efficacy of built sequences. Fitness information could then be fed back into a library of sequences, representing an *in silico* empirically mapped fitness landscape. The fitness landscape comprehensiveness and thus quality of ALife solutions produced by directed evolution, could then be incrementally improved with each iteration of the design-build-test process (Carbonell et al., 2016).

However, the success of directed evolution presupposes a suitably mapped fitness landscape, where such a mapping successfully bridges the fitness landscape disconnect − between a biological agent’s natural fitness landscape and its empirically mapped digital counterpart. Given this, we next review methods for mapping fitness landscapes as a means to effectively guide directed evolution.

## 5 Mapping fitness landscapes

Fitness landscape mapping aims to determine the effects of variations in an organism’s genetic blueprint (genotype), such as sequence changes or design parameters, on the phenotype or performance (fitness) (Greenbury et al., 2022). This is achieved by quantifying how different types of mutations or structural modifications influence overall function in an organism, molecule, or digital organism (Wilke and Adami, 2002). As the sequence length of the genotype increases, the possible variants also increase exponentially, creating a potentially large combinatorial space (Alkan et al., 2011). Mapping such landscapes, therefore, poses a significant technical challenge, not only because of the vast amount of quantifiable data required to adequately sample this space (Vaishnav et al., 2022), but also due to the difficulty of obtaining high-quality, reproducible measurements (Hietpas et al., 2011). Experimental and computational methods must cope with noise, measurement bias, and limited throughput, all of which can obscure the true topography of the landscape (Eling et al., 2019). Despite significant advances that have made in the complete empirical mappings of conceptual representation that maps genotypes to their corresponding fitness values (molecular fitness landscapes), such as that of tRNA (Domingo et al., 2018) and ribozymes (Pressman et al., 2019), enabled by *in vitro* selection and advances in high-throughput sequencing, the intractable size of sequence space limits purely experimental investigations, especially for complete genotype sequences (North et al., 2021). Machine learning is increasingly utilized as a data-driven approach to characterize fitness landscapes and improve directed evolution strategies (Towers et al., 2024). Towers et al. (2024) demonstrated that machine learning models can surpass traditional evolutionary control methods, particularly in highly complex and rugged environments. These models are also practical for laboratory use because they require only population-average data, rather than individual-level measurements, thereby providing a scalable and accessible tool for guiding directed evolution experiments (Towers et al., 2024). This section examines the significance of fitness landscape mapping and the methodologies employed to study fitness landscapes.

### 5.1 Conceptual foundations: why fitness landscapes matter

In evolutionary design, a fitness landscape represents the solution space of a given problem, where the solution representation (genotype encoding) used by the evolutionary algorithm defines the level of detail at which data and features are sampled, represented, and distinguished by the algorithm (granularity) and how the different solutions are connected to each other (topology) of the landscape (Jones, 1995). The specific rule or calculation for assigning a quantitative score that measures the goodness of any possible solution for a given problem (fitness function) serves to select and propagate encoded solutions (genotypes) such that the population of solutions coalesces at optimal regions (fitness peaks) and the algorithm thus generates fit (high-quality) solutions (Eiben and Smith, 2015b). Evolutionary design and optimization has a diverse set of applications, ranging from satellite-antenna design (Hornby et al., 2011), robotic controller design (Doncieux et al., 2015), robotic swarm control (Vásárhelyi et al., 2018), architectural (Kicinger et al., 2005) and building material design (Collins et al., 2016), optimizing ligands against polypharmacological profiles (Besnard et al., 2012) to evolving ALife digital organisms (Wilke et al., 2001). All depend on an evolutionary design algorithm that adequately represents the space of possible solutions as the fitness landscape and then effectively explores the fitness landscape for high quality solutions (Vikhar, 2016). Beyond the digital realm, suitably representing the fitness landscapes of robotic ALife that evolves in physical environments has remained challenging, since the fitness landscape underlying *online* robot evolution corresponds to body-brain couplings that adapt in response to dynamic environments (Doncieux et al., 2015). Thus, with few exceptions (Brodbeck et al., 2015; Hale et al., 2019; Nygaard et al., 2021), most examples of robotic ALife are evolved *offline* in simulation and then physical versions are built for counterpart real-world environments as validation of simulated evolution (Lipson and Pollack, 2000; Jelisavcic et al., 2017).

Similarly, across bioengineering applications, genetic sequences are usually evolved in controlled laboratory environments with the goal of being deployed in uncontrolled (non-laboratory) environments (Cubillos-Ruiz et al., 2021). In such applications, fitness landscapes represent the space of all possible sequences, where specific sequences (genotypes) correspond to desired solutions (phenotypes) (Currin et al., 2015). The relationships between genotype and phenotype in the fitness landscape are the foundation of evolutionary design in bioengineering and, more generally, in natural evolution. For example, RNA enzymes (ribozymes), play critical roles in the RNA world hypothesis, positing that RNA comprises the metabolic architecture of the earliest cells (Pressman et al., 2015). As such, understanding the topography of the underlying fitness landscapes provides valuable insights into molecular evolution, such as how emergent catalytic properties evolve into complex living systems (Kun and Szathmary, 2015).

There have been similar insights for ALife evolved *in silico* and underlying fitness landscapes in simulated evolutionary systems (Franklin et al., 2019), where the concept of a fitness landscape is of critical importance to all directed evolution applications (Castle et al., 2024). For example, comprehensively mapping the protein fitness landscapes of SARS-CoV-2 variants elucidates potential paths of viral evolution thus informing the development of effective anti-SARS-CoV-2 drugs (Flynn et al., 2022). Various independent studies have determined that mostly biological fitness landscapes are rugged, meaning that they can be dynamic and complex with multiple peaks and valleys created by interactions between genes with even single mutations having the potential to cause strong epistatic effects are rugged and dynamic, for example, as demonstrated for viruses (Quadeer et al., 2020), where a high degree of complexity (ruggedness and dynamism) persists across the evolutionary process (Domingo et al., 2023). Despite the seemingly insurmountable complexity of fitness landscapes, recent work indicates that in some biological systems, such as bacterial (Rodrigues et al., 2016), viral (Chéron et al., 2016) and norovirus (Rotem et al., 2018), evolving in response to antibiotic and antiviral treatments, the underlying fitness landscapes can be systematically and quantitatively mapped. For example, Wang D. et al. (2024) found that the fitness landscape of the SARS-CoV-2 virus receptor bindings, evolving versus neutralizing antibodies, can be systematically described through biophysical properties such as antibody binding affinity and protein folding stability.

### 5.2 Simulation-based mapping of fitness landscapes

Simulation-based approaches play a crucial role in evolutionary dynamics by reconstructing or predicting fitness and the examination of adaptive trajectories and evolutionary outcomes landscapes *in silico* (Hoban et al., 2012). Lou et al. (2024) developed a computational method, *Beth-1*, to forecast influenza virus evolution and thus inform efficacious influenza vaccine design. Beth-1 predicts influenza evolution via modeling site-wise (virus genotype segments) mutation fitness, where site-wise fitness dynamics are used to predict the fitness of future influenza variants. Through adaptive estimation of fitness by genotypic site and time, and tracking all advantageous mutations, Beth-1 projects a probable (future) fitness landscape of the virus population. The method demonstrated promising prediction performances in both retrospective and prospective applications using *pH1N1* and *H3N2* virus data, demonstrating a clear advantage of using biological data to map fitness landscapes of influenza viruses. Given such fitness landscape mapping one can feasibly understand the underlying evolutionary dynamics of viruses and use predicted viral evolution to assist in vaccine design. A key limitation to this study was the sequence sample size and sparse evolution time intervals in the data-sets. This impacted the comprehensiveness of the mapped fitness landscape, reflecting that constructed fitness landscapes are only as good as the sampled evolutionary data, meaning predicted evolutionary trajectories may be inaccurate.

Radford et al. (2023) developed a computational method to map the fitness landscapes of synthetic bacterial ribosome (orthogonal tethered ribosome), and permit mutagenesis of nucleotides located in the ribosomal *peptidyl transfer center* (PTC) which is the RNA-based active site in the large ribosomal subunit (50S/60S) that forms peptide bonds (Radford et al., 2023). The ribosome is a macromolecular machine essential for protein synthesis in all organisms (Nissen et al., 2000). Thus understanding ribosome function via mapping underlying its fitness landscape is essential for evolutionary design of synthetic ribosomes for new bio-materials and therapeutics. Radford et al. (2023) reconstructed the fitness landscape of oRiboTs using next-generation sequencing data, where the fitness predictions from analyses of PTC libraries were validated experimentally. Such analyses measured the viability of mutations at individual PTC nucleotides and identified epistatic interactions between positions (nucleobases) within the PTC. This enabled the design of ribosome libraries based on an empirically constructed fitness landscape. Though experimentally validated, mapped fitness landscapes were not used to aid directed evolution for ribosome design. This implores the possibility of feeding back experimentally validated (synthesized) ribosomes to update the fitness landscape and thus guide ribosome evolutionary design.

### 5.3 Empirical mapping of biological fitness landscapes

Empirical mapping aims to experimentally quantify fitness values across defined genotypic variants, providing tangible insight into how mutations shape adaptive landscapes (De Visser and Krug, 2014). Biological fitness landscapes (containing a fixed number of mutational variants of a given molecule or organism) have been partially mapped to study the incidence and distribution of fitness effects of mutations and resultant evolutionary dynamics. For example, Wagner (2023) modeled adaptive evolution on a protein fitness landscape of an *E. coli toxin-antitoxin* system, comprising fitness values for 7,882 antitoxin protein genotypes, and an RNA fitness landscape of 4,176 yeast (*Saccharomyces cerevisiae*) transfer RNA (tRNA) genotypes.

Advances in high-throughput approaches are extending classical deep mutational scanning (DMS) into the single-cell domain, and facilitating direct and high-resolution mapping of genotype–phenotype–fitness relationships (Gantz et al., 2023). By coupling pooled variant libraries with single-cell readouts such as scRNA-seq, these methods capture variant effects across diverse cellular states rather than only bulk averages (Cuomo et al., 2023). For example, Zhao et al. (Qiu et al., 2020) developed *scMPRA*, which links thousands of regulatory variants to cell–type–specific transcriptional outputs within mixed populations, revealing how context shapes fitness effects (Science, 2022). Similarly, Lindenhofer et al. (2025) introduced *SDR-seq*, a single-cell DNA–RNA co-profiling approach that connects endogenous genetic variants to their transcriptional phenotypes at cellular resolution. Together, these technologies move DMS from scalar enrichment scores toward multidimensional, state-aware fitness landscapes that better capture pleiotropy and context dependence in molecular evolution.

Adaptive evolution is modeled as adaptive random walks, starting with a randomly chosen genotype, where the random walk followed genotype variants, changed via point mutations. Wagner (2023) analysis demonstrated that evolvability and fitness enhancing mutations are present in these (protein and RNA) fitness landscapes. However, the analysis only considered a small number of variable sites in mapping the protein and RNA fitness landscapes and relatively few mutations (thus modeling short-term adaptive evolution). This was due to the impracticality of constructing combinatorially complete adaptive fitness landscapes for entire proteins or genotypes for many mutations (long-term adaptive evolution). Though, this limitation holds for adaptive walks of many fitness landscapes mapped from biological datasets (O’Brien et al., 2024). Importantly, conclusions drawn from the modeled short-term adaptive evolution process are not directly comparable across fitness landscapes, because of varying fitness metrics. This is another limitation that continues to frustrate the analysis of evolutionary dynamics across fitness landscapes for various types of genotypes.

### 5.4 Machine-learning-based mapping of fitness landscapes

The enormous size of fitness landscapes corresponding to most genetic sequences has motivated recent work on predictive, data-driven (supervised machine learning) methods to assist in mapping fitness landscapes (Dechant and He, 2021). Such methods essentially interpolate (predict) new data points (functional sequences) given a current library of sequences (training dataset) (Khakzad et al., 2023). Such methods have already demonstrated the efficacy of predicting current and probable future fitness landscapes for entire genotype sequences (influenza *A pH1N1* and *H3N2* virus populations) (Lou et al., 2024), and for directed evolution in protein engineering (Wittmann B. J. et al., 2021). Supervised machine learning has also been applied to map the fitness landscapes of catalytic RNA, which is of special interest since RNA molecules can store genetic information (for example, RNA viruses) and catalyze chemical reactions (for example, ribozymes). Also, the relatively small sequence spaces of RNA makes it suitable for fitness landscape mappings and indispensable for bioengineering applications (Saha et al., 2024). Various supervised machine learning methods using data-driven interpolation and extrapolation over sequence spaces have also been proposed (Saha et al., 2024). Such methods enable prediction of potential evolutionary paths (sets of mutant sequences) between fitness peaks. However, the rarity of functional genotypes means that random sampling of a ribozyme fitness landscape would yield a dataset that is highly biased toward deleterious (non-functional) variants. Thus, supervised machine learning methods are challenged by data bias, since the majority of data will comes from low or moderate fitness landscapes, and where a scarcity of labeled data, that is, sequences with an associated measurement of the target property, continues to significantly limit the predictive capabilities of supervised machine learning applied to fitness landscape mapping (Wittmann B. J. et al., 2021).

Rotrattanadumrong and Yokobayashi (2024) addressed these problems by using *in silico* selection, recombination, and mutation to guide an adaptive walk along evolutionary paths in an RNA sequence space (F1*U ligase ribozyme). This generated a dataset with a more balanced distribution of neutral and deleterious mutants. These data were used to train a deep neural network to predict and identify functional mutational variants that have comparable fitness (relative ligation activity) to a wild-type (a synthesized ribozyme with relatively high fitness). Information about functional variants in distant regions of the fitness landscape was learned by the deep neural network using data acquired from a few mutational steps (starting from the wild-type). This enabled the mapping of the fitness landscape topography around a selected wild-type. The authors discovered an extensive neutral network (van Nimwegen, 2006), a set of genotypes connected by single mutations that sharing the same phenotype (for example, structure, catalytic activity), between the structurally and functionally similar ribozymes (F1*U, F1*U ^m^). This indicated that neutral networks might be common among similar ribozymes, meaning such fitness landscapes could readily be traversed without being blocked by deleterious mutants, that is, evolving populations could travel large mutational distances without detrimental effects on fitness (Knezic et al., 2022). Given this, one could potentially use artificial evolution to engineer ribozymes that adopt a new structure while retaining its function, or acquiring a new function (Portillo et al., 2021). A key contribution of Rotrattanadumrong and Yokobayashi (2024), was integrating information from multiple rounds of *in vitro* selection, so underlying data were not biased solely toward deleterious mutants, thus enabling effective sequence prediction using a deep-learning method. However, supervised approaches require substantial quantities of labeled experimental data, the acquisition of which is both resource-intensive and time-consuming.

The time consuming nature of generating labeled data via experimental means (Rotrattanadumrong and Yokobayashi, 2024), has prompted the application of unsupervised machine learning methods trained on unlabeled sequence data to predict if given sequences will be functional or not. This alleviates the need for labeled datasets while still enabling elimination of non-functional sequences (Riesselman et al., 2018). Such methods include *Generative Adversarial Networks* (GANs) trained on a latent space underlying the sequence space to generate novel functional variant sequences (Ziegler et al., 2023). In such cases, the latent space is an abstract vector space that positions chemically similar sequences near each other (Ding et al., 2019). For example, GANs have successfully generated functional enzyme mutants, with differences of up to 106 point mutations from an original sequence of the *malate dehydrogenase* enzyme (Repecka et al., 2021), where the efficacy of newly discovered sequences can be verified experimentally with *in vitro* methods. For instance the ProteinGAN generated 55 malate dehydrogenase variants, of which 13 (approximately 24%) were both soluble and catalytically active demonstrating experimentally validated functional sequence generation (Repecka et al., 2021). Similarly the PepGAN produced antimicrobial peptides, and 5 of 6 synthesized candidates demonstrated confirmed antimicrobial activity and one peptide achieved a minimum inhibitory concentration of 3.1 µg/mL,which is comparable to ampicillin (Lin TT. et al., 2023). Generative models have also shown promise for novel RNA sequence discovery given their capability to learn underlying patterns and generate novel sequences for testing. For example, *Variational Auto-Encoders* (VAEs), can encode sequences into a low-dimensional latent space and subsequently decode the latent space back into a sequence (Klys et al., 2018). Assuming the latent space accurately represents the fitness landscape, then decoding the latent space assists in mapping high fitness peaks (Iwano et al., 2022). Validation of VAEs in biological sequence design has also been reported, as seen in Hawkins-Hooker et al. (2021), where a VAE trained on luciferase-like oxidoreductase sequences generated novel luxA variants that retained measurable luminescence activity *in vitro* (Hawkins-Hooker et al., 2021). Also, RaptGen employed a variational autoencoder (VAE) architecture trained on Systematic Evolution of Ligands by Exponential Enrichment (SELEX) data to generate RNA aptamers. Several aptamers generated in this study were experimentally confirmed to bind their intended targets with high affinity (Iwano et al., 2022).

Such predictive and generative machine learning methods are invaluable for identifying potentially functional sequences, unlikely to be found via experimental means due to the intractable size of the sequence search space (Biswas et al., 2021). However, as the number of possible combinations grows exponentially with the number of variables, data sparsity and exploration become inefficient *(curse of dimensionality)*, which then pervades evolutionary design and optimization (Eiben and Smith, 2015a), similarly confounds bioengineering applications, since the total number of sequences and possible functional combinations is many orders of magnitude larger than what can be practically synthesized *in vitro*. For example, even representing a small 100 amino acid protein yields a sequence space larger than the number of atoms in the observable universe (Kondrashov and Kondrashov, 2015). Furthermore, biological fitness landscapes are in constant state of flux resulting from the complex interplay between evolving organisms, changing environments and evolutionary phenomena such as epistasis, where the impact of one mutation is dependent on other mutations (Storz, 2018).

Thus, mapping fitness landscapes for the purposes of directed evolution poses major technical challenges due to extremely large amounts of quantifiable data required (Saha et al., 2024). The intractable complexity of biological fitness landscapes highlights the need for universal adoption of canonical *in silico* fitness landscape mapping procedures to ensure consistency in mapping across disparate fitness landscapes (Alvarez et al., 2024). Furthermore, experimental *in vitro* procedures enable experimenters to determine if given mutations, for example, of machine learning generated or predicted sequences, are actually enhancing fitness (Wagner, 2023). For example, bioengineering applications can maintain a digital library of genetically diverse sequence mutants, where the fitness of all mutants has been experimentally evaluated for the purpose of empirically mapping to a fitness landscape (Yang et al., 2024). Hence, suitably mapped fitness landscapes can be used as predictive and prognostic tools enabling one to predict (given trackable mutations) evolutionary trajectories of sequences in given and related fitness landscapes. For example, the SARS-CoV-2 fitness landscape mapping of Wang D. et al. (2024) is hypothesized to be generally applicable to evolving influenza viruses (Gong et al., 2016; Fonville et al., 2014). Similarly, other fitness landscapes mapping underlying virus evolution, have provided insights into the evolutionary dynamics of viruses enabling more accurate prediction of viral evolution (Luksza and Lassig, 2014). Broad accessibility of fitness-landscape datasets is essential because these datasets support model training, benchmarking, and comparative evolutionary analyses (Pitzer and Affenzeller, 2012). Protein and enzyme fitness data are typically deposited in MaveDB, UniProt, and structural archives such as the Protein Data Bank (PDB) (Notin et al., 2023). Complementary kinetic information is available in BRENDA and Expasy ENZYME (Schomburg et al., 2012). Repositories including Rfam, RNAcentral, and miRBase provide standardized frameworks for sequence and structural annotation of RNA and regulatory elements (The RNAcentral Consortium, 2019). Genomic and viral data are distributed through GenBank, GEO, NCBI Virus, and GISAID (Bernasconi et al., 2021). Consistent use of public databases and metadata standards facilitates reproducible, interoperable, and integrative mapping of biological fitness landscapes across molecular systems (Brancato et al., 2024).

Though overall, such fitness landscape mapping studies continue to highlight the disconnect between fitness landscapes empirically mapped from sampled biological data and those that underlie evolving organisms in nature. Given this, we next discuss the fitness landscape disconnect that continues to frustrate the evolutionary design of synthetic counterparts to digital ALife.

## 6 The fitness landscape disconnect: what to do?

In the broad scope of evolutionary ALife there is a fundamental disconnect between the fitness landscapes that inform directed evolution of digital (*in silico*), physical (robotic), and synthetic (*in vitro*) organisms. For example, ALife evolved in simulation and then built as counterpart physical designs cannot continue to evolve their form (morphology) or function (behavior) in real-world environments due to a disconnect between the fitness landscape underlying digital evolution in simulation versus the fitness landscape underlying robotic evolution in a physical environment (Hauser, 2019). Similarly, in bioengineering, there is a disconnect between the artificial fitness landscapes underlying sequences evolved *in vitro* (in laboratory environments), and fitness landscapes underlying sequences evolved *in vivo* (in natural environments) (De Visser and Krug, 2014).

### 6.1 Consequences of the disconnect

Populations (sequences) evolved *in vitro* are rarely functionally transferable beyond specially controlled laboratories to real-world environments (Brooks and Alper, 2021). This limited transferability highlights a broader challenge of generalisation (Walker et al., 2010). Models or experimental systems designed with narrowly defined objectives or task-specific feature sets often capture context-dependent correlations instead of general principles (Kejriwal, 2021). As a result, their predictive or functional performance declines in variable or less-controlled environments (Cavuoto and Nussbaum, 2014). Recent research on general or foundation models aims to identify invariant features across diverse datasets, which may enhance robustness and external validity (Awais et al., 2025). Furthermore, the fitness functions constructed for such experiments are an approximation defined by a specific experiment setup (Luksza and Lassig, 2014). The sheer diversity of fitness definitions for *in vitro* experiments further supports the notion of a disconnect between fitness approximated under controlled experimental conditions and fitness in nature (Livesey and Marsh, 2020). That is, such fitness approximations rarely account for the many additional properties that contribute to fitness in nature. For example, in protein evolution, mutations that improve functional stability or evolvability are considered concomitant with increased fitness (Hie et al., 2024). This fitness function mismatch between controlled laboratory and uncontrolled real-world environments remains a key reason why organisms evolved *in vitro* are rarely transferable to real-world environments for continued evolution.

### 6.2 Empirical insights and partial remedies

Despite the disconnect between empirically mapped and natural fitness landscapes, which will inevitably persist for complex (multi-cellular) organisms due to the enormous complexity of fitness landscapes and confounding factors such as epistasis (Claudia, 2022), suitably (partially) mapped fitness landscapes are still a powerful tool for informing directed evolution for bioengineering applications (Currin et al., 2015). Empirically mapped (computational) fitness landscapes constructed from fitness data (gathered via specially formulated *in vitro* experiments) remain essential for three key reasons. First, a sufficiently large digital library of sequences, derived, for example, via generating with artificial evolution or generative machine learning, *N* mutational variants from a set of initial wild-types, enables researchers to infer (using predictive machine learning) the relative competitive advantage (fitness) per sequence and thus define a suitably mapped fitness landscape (Hie et al., 2024). Second, a sufficiently large sequence library enables researchers to observe the short-term evolution (relatively few generations) of disparate sequences under specific, consistent and controlled laboratory conditions (Yilmaz et al., 2021). Third, such controlled laboratory conditions enable researchers to readily manipulate (direct) and observe the evolutionary trajectories of diverse sequences (in terms of genetic coding and fitness) thereby allowing causal relationships between genetic variation and fitness to be investigated (Van den Bergh et al., 2018).

Considerable progress has already been made in mapping combinatorially complete fitness landscapes corresponding to the complete genotypes of simple single celled micro-organisms such as yeast (*Saccharomyces Cerevisiae*) and bacteria (*Escherichia Coli* (Faure et al., 2022). Such fitness landscapes have then been used as the basis of directed evolution efforts (Wang et al., 2021). In terms of bioengineering entire synthetic (micro) organisms, there are already established directed evolution frameworks operating on entire genotypes. For example, *Adaptive Laboratory Evolution*, is a directed evolution method where microorganisms are cultured in controlled laboratory environments, enabling *in vitro* genotype variation, generational propagation and selection of phenotypes that exhibit improved growth in their environment (Lee and Kim, 2020). For example, one application is the selection of *Escherichia Coli* strains with increased resistance to high temperatures across multiple generations (Tenaillon et al., 2012). Another prevalent example is the genotype-scale directed evolution using *Multiplex Automated Genome Engineering*, which has been applied to develop novel variant organisms such as a new antioxidant producing *E. coli* strain (Wang et al., 2009). More recent approaches use RNA interference and CRISPR/Cas9 systems for genotype manipulation within a single cell, demonstrating effective selection of yeast strains with multiple phenotypes (Si et al., 2017). Thus, suitably mapped fitness landscapes (sufficiently large sequence libraries) facilitate reproducible experiments necessary for continued, progressively comprehensive, fitness landscape mappings that are expected to facilitate the development of new methods for the directed evolutionary design of multicellular organisms (Yi and Dean, 2019).

### 6.3 Toward integrative evolutionary design

Given prevailing unknowns as to how fitness landscapes adapt in concert with changing environments (Yi and Dean, 2019), fitness landscape mappings, at least in the short term, are unlikely to be applicable to all levels of evolution. Even though fitness landscape mappings are currently inadequate for describing complex organism evolution (accounting for the interplay and cyclic feedback between an organism’s genotype, phenotype and environment), the fitness landscape remains an unparalleled concept that succinctly encapsulates molecular evolution, and is thus an indispensable model for evolutionary design in ALife research. Furthermore, significant promise can be observed in recent work that bridges the computational and biological fitness landscape disconnect via mapping large and combinatorially complete fitness landscapes in micro-organisms such as *Escherichia Coli* (Papkou et al., 2023). Such work provides valuable insights into generally applicable evolutionary traits such as demonstrating that fitness landscape ruggedness does not preclude evolving populations from accessing high fitness peaks.

Furthermore, significant promise can be observed in recent work that bridges the computational–biological disconnect by experimentally mapping combinatorially dense fitness landscapes in microbes. For example, a complete five-mutation landscape of the TEM-1 β-lactamase in *E. coli* revealed that sign epistasis makes most mutational routes to high-resistance genotypes inaccessible, yet evolution can still reach global fitness peaks through a few specific adaptive paths (Weinreich et al., 2006). Similarly, large-scale mapping of the green fluorescent protein (GFP) fitness landscape, measuring the function of tens of thousands of variants expressed in bacteria, revealed a narrow, epistasis-rich landscape in which most single substitutions are deleterious, enabling predictive modeling of genotype–phenotype relationships (Sarkisyan et al., 2016). Together, these studies illustrate that ruggedness does not preclude access to high peaks but instead constrains the set of viable trajectories, offering generalizable principles for evolutionary prediction. In this context, success is often defined by achieving near-complete mapping of genotype–fitness relationships, demonstrating reproducible epistatic interactions, and developing predictive models that generalize to unmeasured genotypes or related systems.

Thus, fitness landscape mappings remain invaluable models of a genetic sequence space, enabling insights as to how each sequence corresponds to its activity and can be used to guide the evolutionary design of synthetic agents. The final section of this review thus brings together the highlighted potential of directed evolution methods that suitably balance exploitation versus exploration of the sequence space, with bridging the fitness landscape disconnect via combining *in silico* fitness landscape mapping with *in vitro* sequence fitness evaluation in an iterative design-build-test cycle for *on-demand* evolutionary design of synthetic ALife problem-solvers (agents).

## 7 A bioengineered future: hybrid ALife

The multi-disciplinary fields of synthetic biology (Voigt, 2020) and ALife (Aguilar et al., 2014) are closely related and can be viewed as synthetic and digital counterparts addressing the same goal of deriving new principles and methodologies governing the bottom-up synthesis of living systems on either digital or robotic (ALife) or bioengineering (synthetic biology) experimental platforms. Both fields were founded on the premise of synthesizing either digital, robotic or biological agents in controlled environments to better understand the origins, evolution and development of living systems, learning from *life as it could be*, while addressing the core challenge of consistently and predictably synthesize biological systems with desired problem solving behaviors.

Given unresolved issues with fitness landscape mapping, directed evolution, and appropriate fitness evaluation that continue to confound the bioengineering design-build-test cycle (Carbonell et al., 2016), this section proposes guidelines for future synthetic ALife research that complements such current deficiencies with *in silico* machine learning and *in vitro* fitness evaluation. Specifically, we propose extensions the current diverse array of bioengineering methodologies (Grozinger et al., 2019; Hart and Ferguson, 2019; Szymanski et al., 2023; Baier et al., 2023; Voigt, 2020), into a unified and principled counterpart (hybrid ALife design methodology), similar to what has been proposed for the evolutionary design of physical ALife agents (Hale et al., 2019). As with previous evolutionary design methodologies for simulated and physical forms of ALife, for example, in evolutionary robotics (Doncieux et al., 2015), such a hybrid ALife methodology would follow a design-build-test cycle that includes directed evolution (*exploitation*: improving solution quality), and genetic diversity maintenance (*exploration*: evaluating novel solutions), where a suitable balance between exploration and exploitation enables improved fitness landscape mappings.

### 7.1 Towards a hybrid synthetic ALife methodology

**FIGURE 1:**
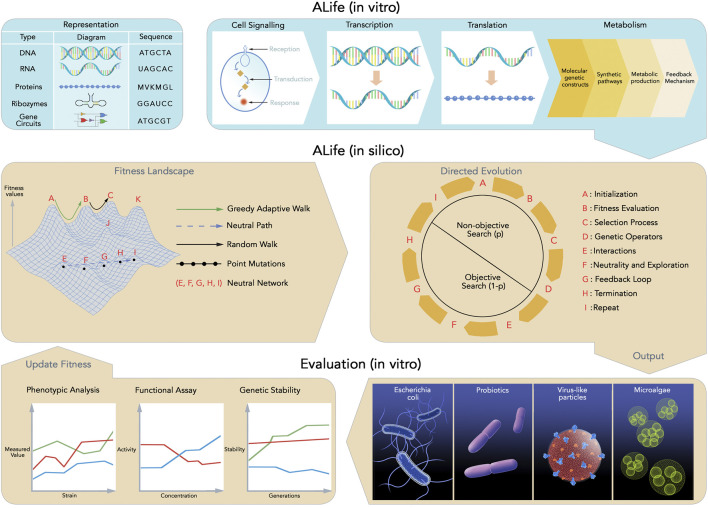
The iterative design process of a hybrid *Artificial Life* (ALife) methodology, combining synthetic (*in vitro*) and digital (*in silico*) evolution. Biological individuals have various potential representations including DNA, RNA, proteins, ribozymes and gene circuits. Directed evolution is applied to biological individuals (sequences), where the viability of each is represented on an *in silico* fitness landscape. Directed evolution combines non-objective (exploratory), objective (exploitative) evolutionary search and predictive machine learning to select individuals for variation. Individuals selected *in silico* are synthesized (varied) and evaluated *in vitro*. Techniques to evaluate the fitness of varied individuals include: phenotypic analysis, functional assays and genetic stability. The fitness landscape is updated, reflecting newly evaluated individuals and used for the next iteration of *in silico* selection, variation, *in vitro* genetic variation and fitness evaluation.

Figure 1 presents our vision of the hybrid synthetic ALife design process. Innovation and novelty in synthetic biology are primarily driven by designers; for example, the *R3C* ligase ribozyme was first created through *in vitro* evolution (Stock and Gorochowski, 2024). Due to the designer-driven innovation approach in synthetic biology, the ability to predict how genetic information (genotype) leads to observable traits (phenotype) is weak and highly dependent on specific conditions, as engineered traits often face evolutionary instability (Wortel et al., 2023). Secondly, standard directed evolution (DE) methods often restrict exploration to genetic variants similar to those already tested, which can trap progress in locally optimal solutions even if other, superior solutions exist elsewhere in sequence space (Wang et al., 2021). Thirdly, the Design-Build-Test-Learn (DBTL) cycle, where ideas are designed, constructed, evaluated, and iteratively improved, remains unbalanced, as the stages after Design (Build, Test, and Learn) lag behind, particularly in terms of data quality and automation (Liao et al., 2022). To address these challenges, we propose an approach that combines both goal-driven (objective) and exploration-driven (novelty, quality diversity) search strategies with adaptive, coevolution-aware constraints and active learning loops (methods where the system iteratively improves based on acquired data). This integration broadens exploration while keeping compatibility with current DBTL workflows. To realize such a design methodology, we propose that future experiments first incorporate advanced forms of directed (objective and non-objective based) evolutionary search (Figure 1, right-center) of *in silico* fitness landscapes (sequence spaces, Figure 1, left-top). Evolutionary search enables the discovery of new sequences using diversity generation and maintenance methods such as *recombination* (Stemmer, 1994), already established in evolutionary design of physical and simulated ALife systems (Eiben and Smith, 2015a), and well known *mutagenesis* methods, often used in bioengineering applications (Currin et al., 2021). This extends previous related work on quantitative computational models that direct the evolutionary design of robotic agents (Brodland, 2015), with objective and non-objective (Nitschke and Didi, 2017) evolutionary search to balance exploration versus exploitation, that is solution *quality-diversity* (Pugh et al., 2016), in the evolving agent design space. In this context, our framework does not replace existing DBTL methodologies. Rather, it adapts these approaches to guide experiment selection in DE-driven synthetic artificial life systems. Critically, beyond a limited set of simulated ALife studies (Gomes et al., 2013; Gomes et al., 2015; Nitschke and Didi, 2017), the impact of hybridized (objective and non-objective) evolutionary search remains untested in the directed evolution of synthetic agents. Consequently, we presuppose that any future synthetic ALife design process will require a careful balance between open-endedness (Packard et al., 2019) and directed (exploitative) evolutionary search as the first key methodological component within an extended DBTL framework.

A second key methodological component of our envisaged synthetic ALife design process, surpassing most ALife design methodologies (Aguilar et al., 2014), but staying inline with current bioengineering design-build-test processes (Carbonell et al., 2016), is experimental (*in vitro*) fitness evaluation. Thus, we advocate controlled laboratory experimental evaluation to validate sequence efficacy (fitness) (Blackiston et al., 2021) (Figure 1, right-bottom), where such experimental validation enables continued, high-precision fitness landscape mapping (Figure 1, left-center). The appropriate validation approach is determined by the class of model predictions and the experimentally measurable aspects of system behavior. Rigorous testing necessitates standardized and quantitative assays, replication, and comprehensive uncertainty analysis to establish confidence in model predictions. Large-scale and automated validation is increasingly performed in robotic biofoundries, where liquid-handling systems and high-throughput measurement platforms enable reproducible and data-rich Build–Test–Learn cycles. This addresses the *reality gap* (Koos et al., 2013) problem that has continued to pervade the design of physical ALife agents with desired functionality (Lipson and Pollack, 2000; Brodbeck et al., 2015; Jelisavcic et al., 2017; Hale et al., 2019). Importantly, both quality-diversity driven evolutionary design and fitness landscape mapping can also be complemented by predictive and generative machine learning (Wittmann BJ. et al., 2021), already established as an effective means for interpolating highly probable novel sequences on evolving fitness landscapes (Brya et al., 2021; Notin et al., 2024; Landwehr et al., 2020). The first and second components of our envisioned synthetic ALife design process will potentially mitigate open issues with directed evolution (balancing exploratory and exploitative evolutionary search with quality-diversity maintenance) and fitness landscape disconnects (validation of evolved solutions with *in vitro* fitness evaluation). This process completes the experimental loop such that model predictions identify candidate experiments, biofoundry testing provides calibrated, high-throughput measurements, and uncertainty-aware learning refines both the landscape model and future design objectives.

The third key methodological component of our envisaged synthetic ALife agent design methodology is integrated predictive and generative machine learning to compute the probable efficacy of novel sequences (Wittmann BJ. et al., 2021). Critically, such machine learning based sequence discovery and validation mitigates the pervasive bioengineering impracticalities of an intractable computational and time expense of *in vitro* validation of all viable sequences. The choice of learning strategy depends on the specific application and model type, which may involve data-driven statistical learners for sequence to function inference or mechanistic and agent-based surrogates that generalize behavioral or ecological dynamics. In each scenario, the Learning phase involves iterative model refinement based on feedback from experimental testing, guided by uncertainty estimation, active learning, and error-driven retraining. This approach completes the DBTL cycle by converting experimental outcomes into updated priors and refined design objectives. All three components extend previous work on current design-build-test processes, where computational design trials translate to counterpart biological trials (Carbonell et al., 2016), for the purpose of providing a unified methodology for synthetic ALife design. An end goal is thus to provide bioengineering and ALife communities with a singular, coherent principled approach for *on-demand* evolutionary design of synthetic (problem solving) agents. Such a methodology would constitute an improved counterpart to current robotic (Doncieux et al., 2015) and simulated ALife agent (Aguilar et al., 2014) evolutionary design methods, where such methods remain *ad hoc* and disparate without a single unifying methodology for ALife design. A coherent evolutionary design process that suitably integrates these three methodological components will be necessary if synthetic ALife agents are to be evolved as solutions to challenging and practical applications.

### 7.2 Applications and controlled environments

Specifically, we anticipate such a hybrid ALife design methodology would most practically be for bioengineering synthetic ALife agents that operate in controlled environments such as indoor farms, factories and manufacturing plants. For example, producing sustainable bio-fuels, plant-based meats and enabling sustainable industry using renewable bio-based raw materials (Voigt, 2020). One can envisage the evolutionary design of synthetic agents (biological organisms) that operate within structured environments. For example, microorganism chemical factories that synthesize bio-fuels and plant-based food (Hillson et al., 2017), but adapt to unexpected errors and changing tasks, or genetically modified organisms bioengineered to mitigate the spread of disease. For example, genetically engineered malaria free mosquitoes that mate with wild populations and spread genetic traits that reduce malaria transmission (Robinson and Nadal, 2025). Such an operational capacity constraint is deemed necessary (at least for the near future), given the enormity of challenges and complexities in bioengineering synthetic ALife agents that would continue to evolve and adapt outside of strictly controlled laboratory environments (Brooks and Alper, 2021). Solving the critical predicament of synthesizing agents that perpetually evolve, adapt and function outside of the laboratory environment, in nature, is tantamount to solving the *reality gap* problem that continues to confounded robotic ALife systems (Koos et al., 2013). Furthermore, controlled environments would enable experimenters to focus their research efforts to evolving agent designs for specific tasks, increasing the likelihood of discovering beneficial sequences in the solution space, leading to the directed evolution of desired agents.

### 7.3 Emerging tools and future directions

Furthermore, in the coming decades it is predicted that increased affordability of genetic sequencing and synthesis will mitigate current experimental screening and validation fiscal and time cost bottlenecks (Robinson and Nadal, 2025). Reduced experiment costs are posited to be aided by emerging plant and animal cell bioengineering tools. For example, *3D Organoids*, have been proposed as *in vitro* models that would eventually replace animals as the experimental platforms in bioengineering research (van Berlo et al., 2021), where such advances are predicted to enable the eventual development of *living computers* that integrate biological and digital components (Robinson and Nadal, 2025). However, if such living computer platforms are to be practical advanced problem solvers, they must be coupled with similarly advanced computational methodologies to direct solution design. Recent advancements in laboratory automation, including robotic liquid handling, high-throughput DNA assembly, microfluidic testing platforms, and integrated biofoundries, are projected to substantially enhance experimental throughput (Ma et al., 2024). These automated systems enable rapid construction and parallel testing of large construct libraries, effectively merging design and validation phases into continuous, data-driven design-build-test-learn cycles (Gurdo et al., 2023). Thus our proposed hybrid synthetic ALife design methodology, combining evolutionary design *in silico* and experimental validation *in vitro*, is a step towards the realization of such living computer systems, which would simply be experimental platforms for the design of synthetic ALife agents.

Our proposed hybrid synthetic ALife design methodology builds directly on earlier ALife work that combined computational design with physical evaluation, most notably in robotics, where evolutionary algorithms were paired with rapid-prototyping technologies to produce and test robots designed *in silico* (Lipson and Pollack, 2000). More recently, analogous principles have been applied in bioengineering: for example, Blackiston et al. (2021) evolved biological robots using an integrated *in vitro* and *in silico* design pipeline. By adapting and generalizing these ideas to the domain of synthetic biology, our framework aims to unify such disparate ALife methodologies and extend contemporary bioengineering design-build-test cycles (Carbonell et al., 2016) into an *evotype* design space (Castle et al., 2021). In doing so, we outline how proven iterative design processes can be leveraged to derive complete genotypes for novel synthetic products (Foo and Chang, 2018), positioning hybrid ALife as a coherent and extensible design paradigm for future synthetic biological systems.
